# Obtention and Characterization of Microcrystalline Cellulose from Industrial Melon Residues Following a Biorefinery Approach

**DOI:** 10.3390/molecules29143285

**Published:** 2024-07-11

**Authors:** Ricardo Gómez-García, Sérgio C. Sousa, Óscar L. Ramos, Débora A. Campos, Cristóbal N. Aguilar, Ana R. Madureira, Manuela Pintado

**Affiliations:** 1 CBQF—Centro de Biotecnologia e Química Fina—Laboratório Associado, Escola Superior de Biotecnologia, Universidade Católica Portuguesa, Rua Diogo Botelho 1327, 4169-005 Porto, Portugal; rgarcia@ucp.pt (R.G.-G.);; 2 CIICYT—Centro de Investigación e Innovación Científica y Tecnológica, Unidad Camporredondo, Autonomous University of Coahuila, Saltillo 25280, Coahuila, Mexico; 3 BBG-DIA—Bioprocesses and Bioproducts Group, Food Research Department, School of Chemistry, Autonomous University of Coahuila, Saltillo 25730, Coahuila, Mexico

**Keywords:** biopolymer, circular bioeconomy, crystalline cellulose, food-waste biorefinery, melon residues, X-ray diffraction

## Abstract

Residual melon by-products were explored for the first time as a bioresource of microcrystalline cellulose (MCC) obtention. Two alkaline extraction methods were employed, the traditional (4.5% NaOH, 2 h, 80 °C) and a thermo-alkaline in the autoclave (2% NaOH, 1 h, 100 °C), obtaining a yield of MCC ranging from 4.76 to 9.15% and 2.32 to 3.29%, respectively. The final MCCs were characterized for their chemical groups by Fourier-transform infrared spectroscopy (FTIR), crystallinity with X-ray diffraction, and morphology analyzed by scanning electron microscope (SEM). FTIR spectra showed that the traditional protocol allows for a more effective hemicellulose and lignin removal from the melon residues than the thermo-alkaline process. The degree of crystallinity of MCC ranged from 51.51 to 61.94% and 54.80 to 55.07% for the thermo-alkaline and traditional processes, respectively. The peaks detected in X-ray diffraction patterns indicated the presence of Type I cellulose. SEM analysis revealed microcrystals with rough surfaces and great porosity, which could remark their high-water absorption capacity and drug-carrier capacities. Thus, these findings could respond to the need to valorize industrial melon by-products as raw materials for MCC obtention with potential applications as biodegradable materials.

## 1. Introduction

In the European Union, food-waste residues (FWR) account for at least 180 M tons, representing 10 to 15% of the total waste generated worldwide, with a huge associated management/treatment cost of up to EUR 140 billion. About 50% of FWRs are semi-treated in a suitable way, accounting 29% for recycling and 20% destined for composting or anaerobic digestion treatments, while the remaining percentage is just disposed in landfills or incinerated [1,2]. Nonetheless, these management strategies must change in the near future and follow the new European Directives that are currently being launched (article 11 and article 5) and are committed to decreasing landfilling to 10%, while assuring sustainable managements of FWR to 65% by 2030 [3,4]. Therefore, the development of suitable strategies for food-waste management under the biorefinery approach plays an important role in increasing sustainability and achieving the challenges that food-processing industries may face in the future [5,6]. The biorefinery approach combines simultaneously different processing treatments on the raw materials to maximize productivity and recovery of several high value-added products. In this context, a biomass-based biorefinery could produce fuels, electricity, and power, as well as several products (i.e., plastics and chemicals), analogously to a petroleum refinery, maximizing the value derived from biomass components and intermediates (i.e., enzymes, bioactive compounds, biodegradable plastics, and nanoparticles, among many other molecules) [7,8]. A biomass-based biorefinery helps to reduce energy consumption and greenhouse-gas emissions, compared to traditional energy-generation plants [9,10]. Biorefineries are expected to play a significant role in the future of energy, biochemicals, and biomaterials generation [10,11]. Currently, fruit-processing industries need a modernization under biorefinery and circular economy concepts, since 30–60% of the total fresh weight of fruit represents food waste (peels, seed, and stems, among others), emphasizing bioresource recovery efficiency, circularity, and valorization of their waste and by-products of a lignocellulosic nature. For instance, these residual biomasses do not yet have a relevant industrial application on a big scale nor in a real waste management case for the obtention of value-added compounds with high industrial interest, losing the opportunities and benefits to develop new businesses, revenue streams, and possible jobs. Instead, they are generally disposed of in landfills as the most accessible treatment, where all their potential natural value is wasted and lost [12,13].

In this matter, melon (*Cucumis melo* L.)-processing industries, within their lineal business production of foodstuffs such as juices, salads, and snacks, among others, generate high amounts of peels as by-products (8 to 20 M tons wasted/year). These melon residues represent biomasses rich in cellulose (20–35%), hemicellulose (10–25%), lignin (5–20%), pectin (15–30%), and proteins (5–15%), among other bioactive compounds [14,15,16]. Such melon peels could be valorized and exploited under the approaches of a biorefinery and a circular bioeconomy, using this biomass as a feedstock for the development of novel eco-friendly and value-added products [17,18]. Hence, an integrative biorefinery strategy will consist of the obtention of multiple different individual compounds from waste biomasses under cascading processing and a zero-waste approach, with the intention of providing more economic and environmental benefits, including not only an industrial melon-processing system but also a processing system for other fruits that is a more sustainable and eco-friendly production system, promoting a circular economy [19,20]. 

In this regard, cellulose, which is the most abundant polymer in nature, is also one of the most prominent constituents of food-waste biomasses. It is arranged by β-(1–4) linked *D*-glycosidic units, which have mainly been applied for paper making, adhesives, textiles, food, and pharmaceuticals due to its outstanding features and properties, such as biocompatibility, biodegradability, renewability, low-cost, and null toxicity. The structure of a cellulose polymer is mainly comprised of highly ordered regions (a crystalline structure) and disordered regions (amorphous structure), contributing to the rigidity and flexibility of fibers, respectively [21,22]. Microcrystalline cellulose (MCC) is one of the most prominent cellulose derivatives and is characterized as a fine, white, tasteless, odorless, purified, and partially depolymerized powder obtained after cellulose hydrolysis [23], having an estimated degree of polymerization (DP) ranging from 70 to 400 glucose units (GUs) [24]. Although the DP is used as a distinctive test, for example, the MCC pharmacopeia is defined by a DP below 350 GUs, compared to DPs on the order of 10 thousand GUs for the original cellulose [25]. The conventional development of MCC involves first the obtention of cellulose polymer from the lignocellulosic raw materials involving mechanical processes, such as grinding and homogenization, through the application of a hot alkali (5–10% NaOH) treatment for hemicellulose and lignin removal. This is followed by aqueous mineral acids hydrolysis at controlled temperatures (80–100 °C), using hydrochloric or sulfuric acids (30–65%) to hydrolyze the amorphous regions of cellulose and finalizing with a bleaching treatment with sodium hypochlorite or hydrogen peroxide to eliminate any remaining color and obtain white polymer fibers, drying and reducing them to a micro-scale level [24,26,27]. This natural polymer is highlighted as a valuable material because of its crystallinity, mechanical properties, high surface area/porosity, and its ability to form hydrogen bonds, forming an organized network that makes it difficult for molecules to pass through [28,29,30]. Thus, its incorporation into composite-materials development is the subject of intense research as a reinforcing and fencing agent, focusing on its water-vapor and oxygen properties, as well as serving as a binder and filler agent in food and medical tablets [31,32,33]. In addition, MCC also has functional properties, such as water absorption, suspension stabilizing, viscosity controlling, and emulsification [34,35,36]. Some studies have shown that MCC can be obtained from lignocellulosic waste on a dry basis, e.g., corncobs and sugarcane bagasse [37], bamboo [36], esparto grass [38], grape stalk [39], lemon seeds [30], pineapple peels [40], rose stems [41], sago seed shell [42], and tea waste [43]. In recent years, biocomposites and microcellulose materials have been relevant natural key compounds for researchers and industries because such materials are, indeed, better and sustainable alternatives due to their renewability and biodegradability compared to traditional petroleum products, which could allow for a less negative environmental impact [44,45].

However, to the best of our knowledge, studies on the valorization of cellulosic materials from melon-peel by-products have not been extensively explored or reported yet. Thus, to further increase the overall value of this melon biomass, a full-detailed structural characterization of melon-peel residues after pectin extraction was carried out to obtain MCC. This research study aimed to explore melon-peel by-products as a bioresource to recover value-added compounds, such as cellulose, in the context of a biorefinery as a suitable strategy for their integral valorization following an MCC obtention process and comparatively examined its extraction yield, psychochemical structure, and morphology.

## 2. Results

### 2.1. Chemical Composition and Structural Changes of Lignocellulosic Melon By-Products (LMB) after Pectin Extraction

The chemical composition of the melon peel (LMB) before and after the pectin extraction process is depicted in Table 1. As can be seen, the content of protein, fat, and ash in RHCl, RCA, and RTA decreased during the hot-acid treatment, since most of these compounds are solubilized/dragged into the liquid medium during the treatment, demonstrating an increase in the solubility of these molecules by the combination of temperature and acidic environment. The hydrothermal conditions allow for a better affinity of these compounds to the liquid medium. Therefore, the total fiber contents observed were from 37.10% in LMB to 49.60%, 55.51%, and 77.71% for RCA, RTA and RHCl, respectively. Dietary fiber is classified as water-soluble (pectin) and water-insoluble fibers like cellulose, hemicellulose, and lignin.

In this regard, LMB showed a composition of 24.24% cellulose, 12.05% lignin, and 11.04% hemicellulose, which was incremented after pectin extraction, ranging from 40.13 to 58.84%, 21.33 to 27.50%, and 18.39 to 29.46% for cellulose, hemicellulose, and lignin, respectively (Table 2). These increments were observed since the pectin was successfully removed from the melon-peel by-products (data not shown) by a combination of elevated temperature and vigorous thermal agitation, which resulted in the breakdown of hydrogen bonds and other intermolecular interactions. These interactions enhance the dissolution of pectin in water [46], resulting in a lignocellulosic melon biomass free of impurities for the extraction of microcrystalline cellulose (MCC). The cellulose contents are comparable to that of some different sources, for example, natural wood (48.30%) [47], and are higher than banana peels (23.37%) [48], citrus peels (34.80%) [49], and mango seed (39.00%) [46]. The cellulose, hemicellulose, and lignin contents are high owing also to the removal of pectin from the initial sample (LMB), which concentrates the structural components. The removal of extractives could allow for an easier and faster cellulose extraction from the plant matrix with less usage of corrosive agents. Also, lignin seems to be slightly removed during pectin extraction, helping in the extraction of purified cellulose.

### 2.2. Microcrystalline Cellulose (MCC) Extraction and Structural Composition

**Figure 1:**
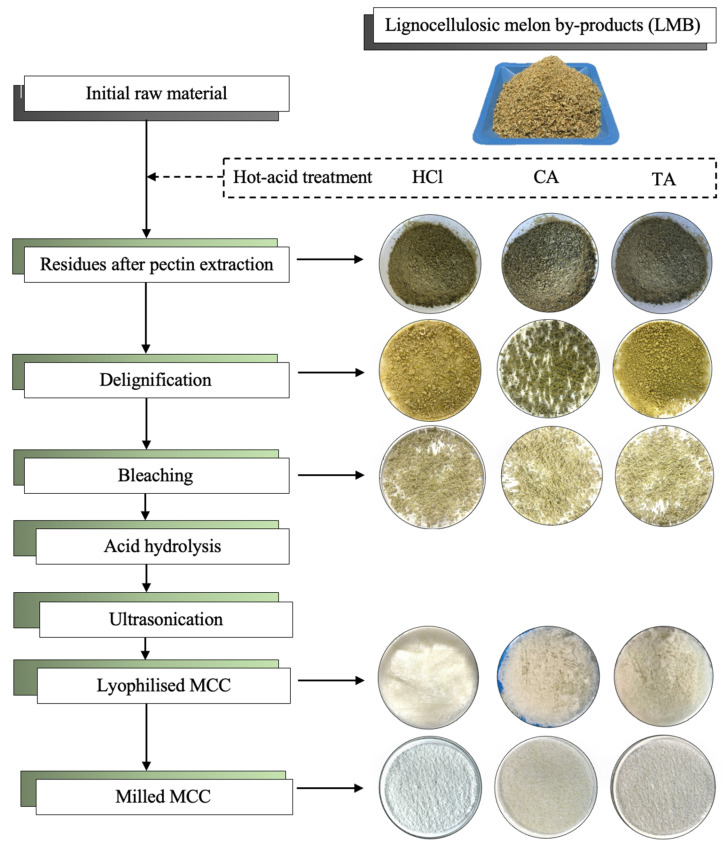
Flow diagram of the process to obtain microcrystalline cellulose (MCC) from melon residues through a thermo-alkaline process. HCl: hydrochloric acid; CA: citric acid; TA: tartaric acid.

As can be appreciated in Figure 1 the untreated dry LMB presented an initial light yellow–brown color that changed to dark green after hot-acid treatment. Then, it was yellow-like after the delignification process by the thermo-alkaline method and became white after acid hydrolysis. The change in color of the LMB from dark green to white indicates the effective removal of lignin from the melon fibers. The mass yield of the LMB through each step of both extraction processes indicates the successful recovery of the biomass throughout the obtention of MCC (Table 3). Samples treated by the thermo-alkaline process obtained the highest value of bleached fibers for RHCl (53.67%), followed by RCA (49.81%) and RTA (43.39%). However, the RCA sample treated by the traditional process (4.5% NaOH) had the highest bleaching yield 77.84%, followed by RTA (77.04%) and RHCl (71.59%). After freeze-drying, cellulose biomass, visualized as white-spongy fibers, was obtained (Figure 1) In this step, the maximum yields obtained were 13.04% and 23.81% for HCl by thermo-alkaline and traditional processes, respectively. These yields are similar to some already described in the literature for the acid hydrolysis of cellulose from other sources, i.e., 12.10% from tea waste [43], 14.60% from lemon seeds [30], and 26.10% from corncobs [39], but lower compared to the 27.24 and 32.12% from corn and sugarcane, respectively [37], and the 53.60% from bamboo [36]. Factors such as concentration of acid, temperature, and reaction time are critical parameters to keep under constant control during acid hydrolysis because such hydrolysis, in many cases, depends on the plant material. For example, high acid concentrations can destroy cellulose fibers. For this, it is preferably performed under mild conditions, at a low temperature (<90 °C) and low acid concentrations (<70%), to avoid cellulose deterioration, but in other cases, these conditions are not able to complete the dissolution of the amorphous cellulose region. In our particular case, all the conditions used in this study were implemented based on these reported factors, employing 80 °C and 30% acid concentration as improved conditions for melon-fiber hydrolysis. Therefore, to the best of our knowledge, these results on melon MCC were, for the first time, obtained from melon-peel residues after pectin extraction, within the framework of the biorefinery approach. Table 4 shows the structural composition, ash, and moisture content of MCC obtained from the treatment of melon-peel residues (RHCl, TCA, and RTA) by thermo-alkaline and traditional processes. Microcrystalline cellulose RHCl had the highest content in the cellulose (48.34% and 55.03%), followed by MCC-RTA (46.31% and 52.51%) and MCC-RCA (44.89% and 51.40%). The lignin content was highly reduced in all the MCC samples, reaching values below 4.00% and even lower than 3.00% by thermo-alkaline and traditional processes, respectively. This indicates that the mixture of NaOH and H_2_O_2_ effectively removed most of the lignin present in the untreated LMB fibers. The moisture content is an important parameter that should be considered when determining if a natural material is an appropriate filler for polymer composites, as it influences the functionality and mechanical properties of MCC. The permissible maximum moisture content is estimated at <7.00%, according to the United States Pharmacopoeia (USP) since too much humidity could negatively affect the fibres and the properties of the polymer composites or material where MCC will be incorporated [38].

Thus, these melon MCC samples obtained by both processes that presented a lower moisture content (3.90 to 5.23%) are within the limit recommended and are comparable to the 5.80% reported for the MCC from *Posidonia oceanica* algae that was obtained by acid hydrolysis. Also, the ash content was comparable to the 2.00% for pineapple peels [40] and lower than the 8.80%, 11.85%, and 14.30% for walnut shell, corncob, and sugarcane bagasse, respectively [37].

### 2.3. X-ray Diffraction

**Figure 2:**
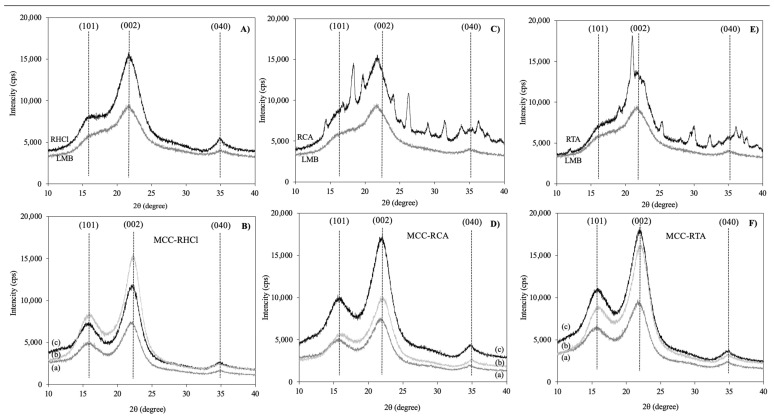
X-ray diffraction patterns of lignocellulosic melon by-products (LMB), melon residues after hot-acid treatment with (**A**) hydrochloric acid (RHCl), (**C**) citric acid (RCA), and (**E**) tartaric acid (RTA) and their microcrystalline cellulose (MCC) (**B**,**D**,**F**), respectively, obtained through the (a) thermo-alkaline, (b) thermo-alkaline double bleaching, and (c) traditional treatments.

Figure 2 shows the diffraction profiles of the LMB, melon residues (RHCl, TCA, and RTA), and MCC samples obtained by both processes. The untreated LMB fibers had a less-concentrated cellulose (also see Table 2), showing thicker and fewer defined peaks, while the RHCl showed a pattern typical of cellulose and an increment in the intensity of the peaks, suggesting that the cellulose became more available after the hot-acid treatment with this acid (Figure 2A). As for the RCA and RTA samples, they showed irregular profiles with small peaks, which could be attributed to the presence of some salts or minerals embedded in the MCC of these residues derived from the hydrolysis with citric and tartaric acids (Figure 2C,E, respectively). The individual diffraction patterns of the MCC from the alkaline treatments and the untreated LMB fibers occurred at 2θ angles of 15.70, 22.31, and 34.98° and can be ascribed to the crystallographic planes (101), (002), and (040), respectively (Figure 2B,D,F), which are consistently characteristic of Type I cellulose (native cellulose) [29]. All related diffraction angles in all the samples from delignification to the bleaching processes might indicate that the cellulose kept its structure intact. In this case, the diffraction peak at 22° was shown to be sharper, and its intensity increased as well, which indicates an improved crystallinity in the structure of MCC regarding the starting material (LMB). For example, all the MCC samples obtained from RHCl, RCA, and RTA showed an intensity increment in their profiles and better-defined peaks (without impurities) after the bleaching step (Figure 2B,D,F), evidencing the crystalline nature of the treated melon cellulose fibers. The presence and defined patterns in the MCC samples could suggest that the chemical process did not alter the crystalline structure of the cellulose fibers. The cellulose Type I can be altered to dissimilar polymorphs by an alkaline treatment (NaOH) known as mercerization, and the cellulose type II chains have an antiparallel arrangement with the ability to produce improved structures. It is fundamental to know how the structure, morphology, and crystallinity of the melon fibers are transformed after each treatment. The degree of crystallinity (DC) was determined for the untreated LMB, melon residues (RHCl, RCA, and RTA), and their respective MCCs (Table 5). The DC was calculated from the height ratio between the intensity of the crystalline peak and the total intensity of the non-crystalline peak using the equation reported by Segal et al. [50] (DC = (I_200_ − I_am_)/I_200_), where DC is crystallinity, I_200_ is the maximum intensity of the peak, and I_am_ is the intensity of diffraction of the non-crystalline material. The DC increased after the hot-acid treatment, starting with 31.82% for the LMB to 42.35–47.75% for the melon residues. Also, the DC of the MCC samples after one bleaching treatment increased (47.03–52.01%), but the values were slightly lower when compared with the traditional process (54.80–55.07%). These results could be attributed to the higher concentration of NaOH used for the traditional method (4.5%) than the thermo-alkaline one-bleaching step (2%), making more efficient the solubilization of the lignin and, consequently, giving an increment on cellulose crystallinity [29,47]. After the double-bleaching step, a higher value of DC was obtained (51.51–61.94%), representing a higher percentage than the traditional process (54.80–55.07%). These results could be attributed to the extra removal of the non-cellulosic components that may still be linked in the amorphous regions of the MCC. The DC of the bleached MCC from the melon residues was in the range of previous reports for other vegetable materials, i.e., 22.20–56.20% from rose stems [51], 44.00% from sago seed shell [42], 62.00% from palm seeds [52], and 62.50% from bamboo [45]. Diverse researchers have described those drastic conditions of acid concentration and time. They not only hydrolyze the amorphous regions of cellulose but can deteriorate the crystalline regions, resulting in a lower-quality MCC [38,42]. In this respect, the melon MCC samples obtained seemed to not be affected by the conditions implemented in this study and fell within the crystallinity range of the commercial MCC, which is in the range of 55–80%.

### 2.4. FTIR Analysis

**Figure 3:**
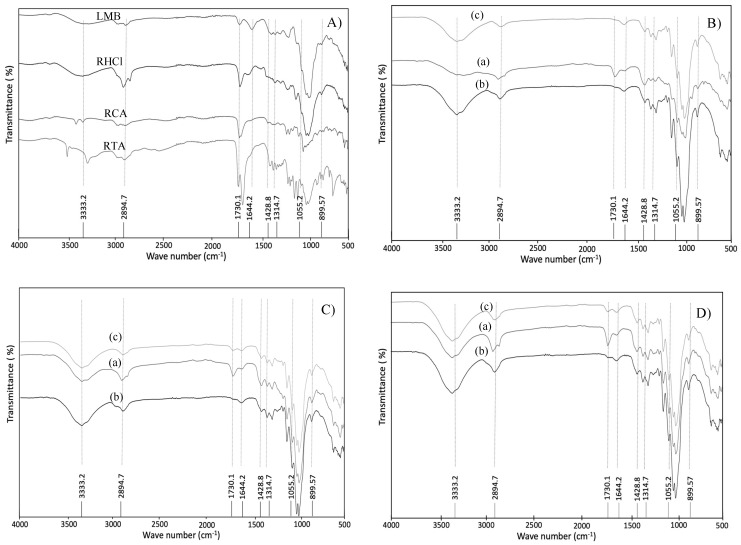
Spectra of (**A**) lignocellulosic melon by-products (LMB) and melon residues after hot-acid treatment and microcrystalline cellulose (MCC) from (**B**) RHCl, (**C**) RCA, and (**D**) RTA through the (a) thermo-alkaline, (b) thermo-alkaline double bleaching and (c) traditional treatments.

The spectra of LMB, melon residues (RHCl, TCA, and RTA), and MCC samples are shown in Figure 3. During the transition from macro- to micro-materials, the alterations are controlled by variations in the hydroxyl and carboxyl zones and those related to the lignin structure [51,52]. In the FTIR spectrum of LMB, melon residues, and MCC samples (Figure 3A–D), the peak positioned at 3333.2 cm^−1^ falls inside the range of 3335–3350 cm^−1^, which corresponds to the stretching vibrations O-H in cellulose [42]. The increase in the peak suggests an increase in cellulose composition and removal of lignin polymer after acid hydrolysis. Harini and Chandra Mohan [37] isolated micro- and nanocrystalline cellulose from different plant-based sources, such as walnut shells, corncobs, and sugarcane bagasse, and also reaffirmed the same behaviors in the intensification of peaks between 3500 and 3200 cm^−1^ associated with the removal of the lignin and, subsequently, giving rise to highly crystalline cellulose fibers. Also, the increase in the intensity of this absorption band after acid hydrolysis could be attributed to the elimination of the amorphous components, increasing hydrogen bonding between the cellulose chains.

**Figure 4:**
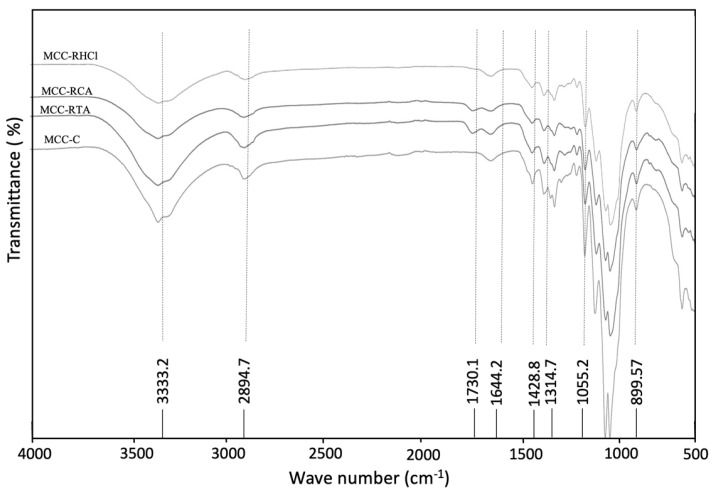
Spectrum of microcrystalline cellulose (MCC) obtained by thermo-alkaline double-bleaching process from melon residues compared to commercial microcrystalline cellulose (MCC-C).

The peak between 2850 and 2900 cm^−1^, positioned at 2894.7 cm^−1^ in all the spectrum of the melon samples corresponds to C-H stretching vibrations [40]. The band observed at 1730.1 cm^−1^ originated from the acetyl and ester groups in the hemicellulose or the carboxylic acid groups of the phenolic compounds linked to lignin, and the peaks at 1450–1600 cm^−1^ correspond to stretching structures of the aromatic groups of lignin [53]. Moreover, the small peaks ranging from 1100 and 1500 cm^−1^ were attributed to proteins [43]. The peak at 1730.1 cm^−1^ was present in the spectrum profile of the LMB, which was intensified in all the residues (RHCl, RCA, and RTA) by the hot-acid treatment. After the one bleaching step, the peak is still present, indicating the presence of hemicellulose in the MCC samples. However, in the spectrum of the MCC samples obtained after double bleaching and the traditional process, the hemicellulose peak disappeared, showing the elimination of hemicellulose from melon MCC during chemical extraction. This peak, observed at 1730.1 cm^−1^, is related to the C=O bonds of unconjugated ketones present in the hemicellulose [54]. This result could indicate that the thermo-alkaline treatment by autoclave 2% NaOH was more efficient in removing hemicellulose from melon fibers than the traditional process. The peak at 1600 cm^−1^ in the spectrum of the MCC samples was attributed to the C=C bond extending from the aromatic ring of lignin, and the peaks near 1055.2, 1314.7, and 1428.8 cm^−1^ were associated with C-O stretching, C-O-C asymmetric stretching, and C-H oscillating vibrations of cellulose, respectively, that appeared in all of the spectra and were increased during the extraction process [33,34,55]. The peak at 899.57 cm^−1^ represents the glycosidic deformation -C1-O-C4, which is characteristic of the β-glycosidic bond of cellulose [30]. This peak showed the typical characteristics of a cellulose structure, and its existence was normally observed in the FTIR spectrum of microcrystalline cellulose obtained with chemical methods involving alkaline-bleaching acid hydrolysis from lemon seeds [30], esparto grass [38], corn, grape, pomegranate, and strawberry [40] as well as in the commercial MCC (Figure 4).

### 2.5. Morphology Analysis by SEM

**Figure 5:**
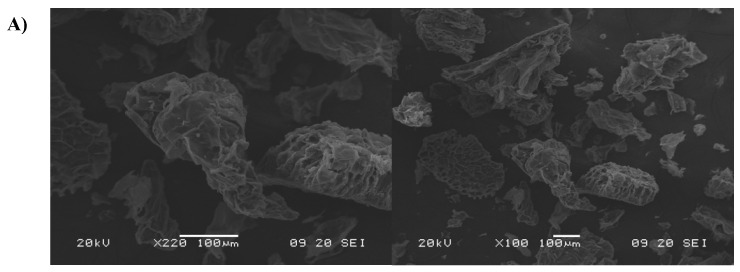

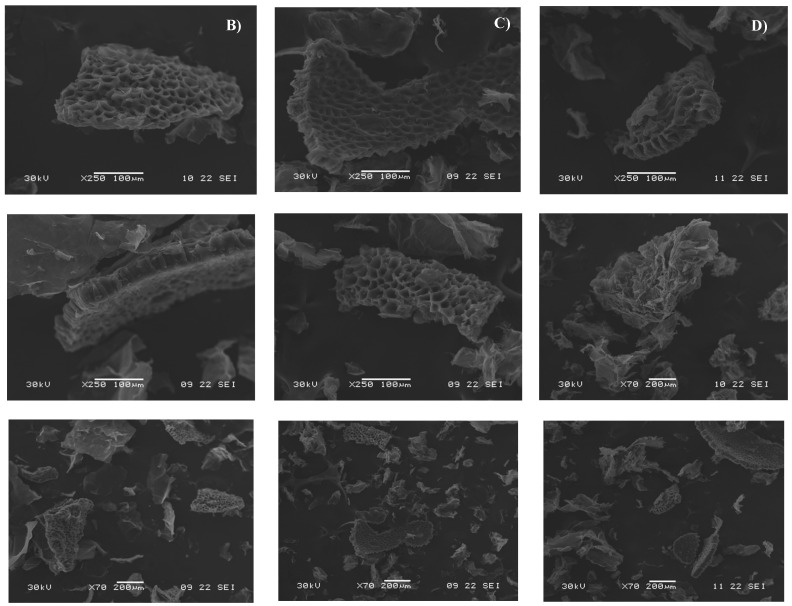
SEM micrographs of (**A**) LMB and freeze-dried MCC from (**B**) RHCl (left-column), (**C**) RCA (middle-column), and (**D**) RTA (right-column) through the thermo-alkaline double-bleaching treatment (white bar represents the scale; 100 or 200 μm).

The morphologies of the LMB and MCC samples are shown in Figure 5. The structure of LMB (Figure 5A) presents a well-closed and agglomerated structure, with a protective layer covering the grooves of the fiber structure. In addition, the untreated LMB sample had pectin, globular wax particles, and extractives, in addition to lignin and hemicellulose. Significant alterations in morphology were observed in the MCC samples obtained from RHCl, RCA, and RTA (Figure 5B–D, respectively), in which the MCC became more exposed after two bleaching treatments, due to the effective removal of the lignin matrix, and separated into individual fibers, showing microcrystals formation with numerous micropores, cylindrical shapes, and fissured and rough surfaces. This was probably produced by the penetration of the H_2_SO_4_ acid solution into the amorphous regions of cellulose during the acid hydrolysis process and the breakdown of the β-1,4-glycosidic linkage between the cellulose repeating units and, subsequently, undergoing fragmentation to form the smaller size of cellulose microcrystals [56]. Microcrystalline cellulose is a particle of hydrolyzed cellulose, comprising a very large amount of cellulose micro-crystals, together with amorphous areas. Micro- and nano-porosity are outstanding characteristics with potential in pharmaceutical and biomedical applications for tissue engineering and biomaterial development [54,56]. Microcrystalline cellulose with high micro- and nano-porosity could be useful as a potential active site for cell growth and blood-vessel invasion, as well as being a tablet filler, a binder–disintegrant, and a drug carrier due to their large surface area and high moisture-retaining property [44]. The possibility of using this porous material as a sustainable functional additive/ingredient is a current investigation trend for the development of hydrogels or pharmaceutical tablets for drug delivery. For efficient drug delivery, pharmaceutical tablets must disintegrate in a short period, without delay, so that the liberated active drug is available for dissolution and, hence, immediate absorption [57]. For example, Pachuau et al. [58] extracted and used MCC from *Ensete glaucum* with 53.41% crystallinity and high porosity for tablet formulation, highlighting its ability to produce adequately hard, yet rapidly disintegrating, tablets, which was attributed to the swelling of its particles and a consequent decrease of the bonding forces holding them together. Therefore, melon peels could be applied as a raw material with low cost for the obtention of MCC, which could be an emerging prototype of an engineered material with a great application because of the properties shown and associated with its potential biodegradability, biocompatibility, low toxicity, and excellent mechanical properties.

## 3. Materials and Methods

### 3.1. Chemicals

All reagents used in this study, such as citric acid, *D*-(+)-glucose, *D*-(+)-xylose, *L*-(+)-arabinose, hexane, hydrochloric acid (HCl), hydrogen peroxide (H_2_O_2_, 30%) sodium hydroxide (NaOH), sulfuric acid (H_2_SO_4_, 98%) and tartaric acid, were purchased from Sigma-Aldrich (Sintra, Portugal).

### 3.2. Biorefinery Approach: Deconstruction of Melon-Peels Biomass in Different Fractions and Value-Added Compounds

**Figure 6:**
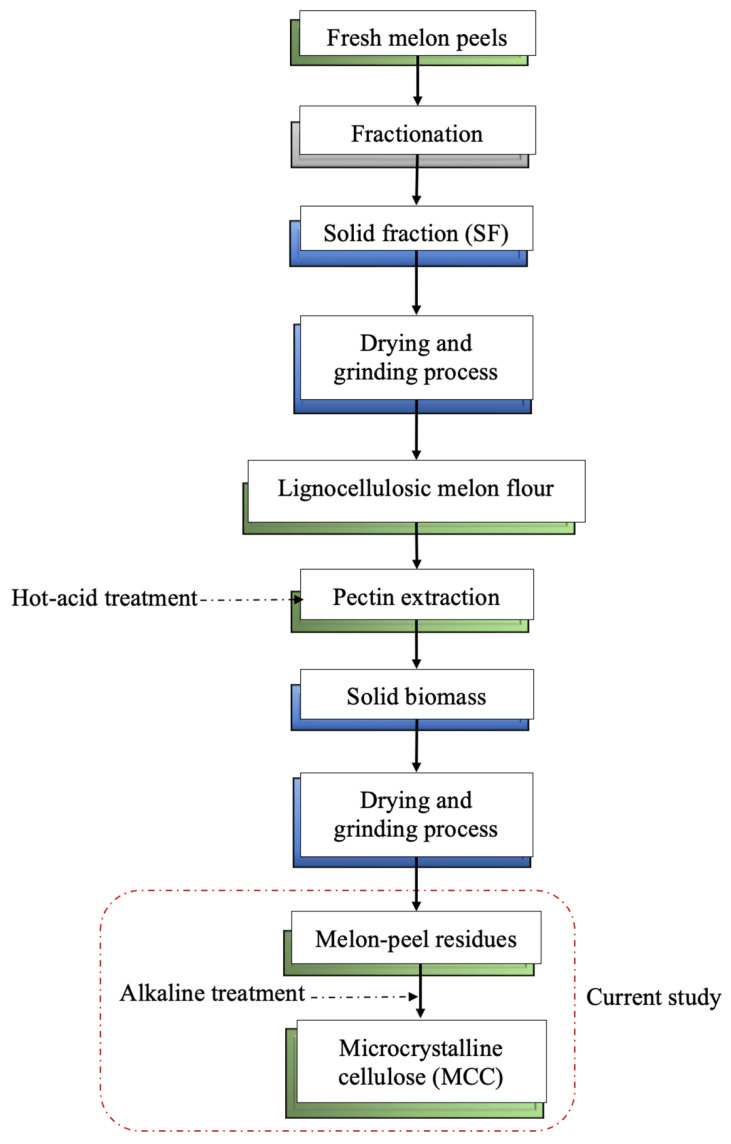
Process flow chart for melon-peel by-product valorization under biorefinery approach for the obtention of different value-added ingredients.

Three different melon-peel residues were obtained after the hot-acid extraction of pectin using hydrochloric acid (HCl) and citric and tartaric acids (CA and TA, respectively). The processing of melon peels (on a fresh weight basis) was handled as described previously by Gómez-García et al. [17]; the melon peels were fractionated using a commercial juicer (HR1869/8, 900 W, Philips, Eindhoven, The Netherlands) to separate the solid fraction (SF) from the liquid. The SF was collected and pressed using a muslin cloth to remove the remaining liquid and then dried in an oven dryer at 55 °C for 48 h. The liquid fraction was used for cucumisin enzyme recovery, as previously reported by Gómez-García et al. [59]. Afterward, the dried SF was ground using a coffee machine (TSM6A011W, Bosch, Munich, Germany) to obtain a fine powder with an estimated particle size no bigger than 250 μm and measured with a sieve of 20 cm in diameter, with mesh sizes of 250 μm. This fraction was called lignocellulosic melon by-products (LMB). Pectin extraction from LMB was carried out in acidified (pH 2) hot distilled water at 90 °C and 120 rpm for 60 min with a solid–liquid ratio of 1:50. Then, the sample was filtered using two layers of muslin cloth, and the solid particles were collected and dried at 55 °C for 24 h. Later, the dried solids were ground as described above and labeled as RHCl, RCA, and RTA residues from hydrochloric, citric, and tartaric acids treatment, respectively. The residues were stored in plastic bags, avoiding humidity and sunlight. The flow chart for the melon-peel fractionation process is shown in Figure 6.

### 3.3. Extraction of Cellulose from Melon-Peel Residues

#### 3.3.1. Traditional Method

An alkaline traditional treatment with NaOH (4.5% *w*/*v*) was carried out on the dried melon residues (RHCl, RCA, and RTA) with a solid–liquid ratio of 1:10 for 2 h at 80 °C under agitation at 200 rpm in an orbital shaker (MaxQ 6000, Thermo Scientific, Waltham, MA, USA). After this, the reaction was cooled in an ice bath (15 min) and then vacuum-filtered using two layers of muslin cloth and washed until elimination of the dark alkaline liquor and attaining neutral pH. The solids were oven-dried for 18 h at 55 °C and ground (particle size ≤ 250 μm). The dried residues were bleached according to the method reported by Ventura-Cruz et al. [41], with a mixture (ratio 1:1) composed of NaOH (5% *w*/*v*) and H_2_O_2_ (16% *w*/*v*) and a solid–liquid ratio of 1:40 for 1.5 h at 55 °C under agitation at 200 rpm in an orbital shaker. The hydrolyzed solids were carefully filtered using a muslin cloth and washed two times with deionized water. The solid materials were oven-dried for 12 h at 55 °C and ground (particle size ≤ 250 μm).

#### 3.3.2. Thermo-Alkaline Method

Melon-peel residues were subjected to a thermo-alkaline treatment in a pressurized autoclave with NaOH (2% *w*/*v*) in a solid–liquid ratio of 1:10 for 1 h at 100 °C. The hydrolysate was cooled (15 min) in a water bath and then vacuum-filtered using two layers of muslin cloth, and the remaining solids were washed with distilled water until the elimination of the dark alkaline liquor and reaching neutral pH. The solids were oven-dried for 12 h at 55 °C and ground (particle size ≤ 250 μm). The bleaching step was carried out twice as described above in Section 3.3.1.

### 3.4. Microcrystalline Cellulose (MCC) from Cellulose of Melon Residues

The MCC was obtained by the acid hydrolysis of the bleached cellulose obtained from each melon residue using H_2_SO_4_ (30% *w*/*v*) at a solid–liquid ratio of 1:20 for 2 h at 80 °C under agitation at 200 rpm in an orbital shaker. After this, the mixture was diluted 10-fold with water at room temperature to stop the reaction and vacuum-filtered using a muslin cloth. The hydrolyzed solids were washed with deionized water until the excess of acid was removed. Afterward, the resulting mixture was put in an ice-water bath and sonicated at 70% intensity in a VCX 130 ultrasonicator (Sonics & Materials, Newtown, CT, USA) for 5 min. The colloidal sample was stored at −80 °C until its freeze-drying process. The yield of MCC extraction (YMCC) was calculated according to Equation (1). The graphic flow chart for the obtention of MCC from melon-peel residues is displayed in Figure 1.

(1) $$\%YMCC=\frac{mass\;of\;the\;freeze-dried\;MCC\;(g)}{mass\;of\;the\;melon\;residues\;after\;cellulose\;bleaching\;(g)}\times 100$$

### 3.5. Chemical Characterization of Melon-Peel Residues and Cellulose Samples

The content of dry matter (DM), moisture, ash, protein, dietary fiber, and carbohydrate constituents of the samples were made in triplicate according to standard procedures (AOAC) and described by Gómez-García et al. [17] as follows.

#### 3.5.1. Dry Matter and Moisture Contents

The dry matter was calculated using the AOAC (1990) procedure [17]. The moisture content was calculated by drying the melon samples (1 g) at 105 °C for 24 h until constant weight (AOAC, 1997). The results were presented as a percentage of %DM.

#### 3.5.2. Ash Content

The ash was measured by carbon removal of the dry residue samples from Section 2.2, which were incinerated in a muffle furnace at 550 °C for 24 h (AOAC, 1980). The total ash content was expressed as a percentage of %DM [17].

#### 3.5.3. Protein Content

The total protein content of the melon samples was determined by the Kjeldahl method (AOAC, 1997). The protein was estimated using the conversion factor of 6.25 for vegetables. The data were expressed as a percentage of %DM [17].

#### 3.5.4. Determination of Structural Carbohydrates Composition

The extractives, structural carbohydrates (cellulose and hemicellulose), and lignin of the melon residues and samples were quantified according to a laboratory analytical protocol reported by Sluiter et al. [60]. Briefly, 3 mL of H_2_SO_4_ (72%) were added to a glass tube containing 0.3 g of the sample and incubated at constant agitation (120 rpm) in a shaking water bath at 30 °C for 1 h. The suspension was diluted to a final concentration of 4% by adding distilled water and then subjected to incubation for 1 h at 121 °C. The hydrolyzed sample was cooled at room temperature and later vacuum-filtered (Pyrex^®^ 50 mL M crucible, Corning, Inc., Corning, NY, USA), and the solid residues were used for gravimetric analysis of acid-insoluble lignin. The filtrate was used for acid-soluble lignin and sugar quantification. The chromatographic separation of the sugars was carried out using Beckman Coulter HPLC equipment coupled to an IR (K-2301) (Knauer, Berlin, Germany). The aliquots were filtered (0.45 μm) into a glass vial. Then, 30 μL of the sample were analyzed using an Aminex HPX-87H column (Bio-Rad, Hercules, CA, USA) operated at 40 °C with 5 mM H_2_SO_4_ as a mobile phase at a constant flow of 0.6 mL/min for 30 min. Data acquisition and analysis were accomplished using Clarity software (Version 5.0.5.98 by DataApex Ltd., Prague, Czech Republic). The peaks were examined and quantified using a calibration curve of each sugar standard (glucose, arabinose, and xylose) by comparing the retention time and areas. The total dietary fiber in each fraction was evaluated using the enzymatic-gravimetric method described by Lee, Prosky, and Vries [61] and later reported in our previous work [17]. Three independent analyses were performed for each experiment.

#### 3.5.5. Fat Content

The total fat content was calculated using the Soxtec method (Soxtec^TM^ 8000, Foss, Hillerød, Denmark). Fat was extracted from 1 g of the sample packed in a cellulose thimble with 60 mL of hexane at 90 °C for 3 h. The examination was made in triplicate, and the results were presented as a percentage of %DM.

#### 3.5.6. Total Carbohydrates

The total carbohydrate content was calculated by the difference of the mean values, as follows: [100 − (%moisture + %ash + %protein + %fat)].

### 3.6. Powder X-ray Diffraction (PXRD)

Tests were performed with a Rigaku MiniFlex 600 diffractometer with Cu kα radiation analyzer (Akishima-shi, Tokyo, Japan), with a voltage of 40 kV and a current of 15 mA (3° ≤ 2θ ≥ 60°; step of 0.01 and speed rate of 3.0°/min). The degree of crystallinity (DC) was calculated as the ratio of the areas under the crystalline peaks to the total area [62].

### 3.7. FTIR

Tests were performed in a spectrometer (Spectrum 100 Perkin Elmer FTIR-ATR, Waltham, MA, USA). The spectra were collected in the range of 500 to 4000 cm^−1^, with a resolution of 4 cm^−1^ and 64 scans per sample [62].

### 3.8. Scanning Electron Microscopy (SEM)

The morphology of melon MCC was evaluated by scanning electron microscopy (SEM) using a JEOL-5600 LV microscope (Tokyo, Japan). Briefly, a small amount of freeze-dried sample was placed over an observation stub covered with double-sided adhesive carbon tape (NEM tape; Nisshin, Japan) and then coated with gold/palladium (Au/Pd) using a Sputter Coater (Polaron, Bad Schwalbach, Germany). SEM was operated in high-vacuum mode using an accelerating voltage of 15–20 kV [62].

## 4. Conclusions

Currently, there exists an exigent need not for only more sustainable and natural foods but also for biofuels, biochemicals, and biomaterials, targeting biodegradability and renewability. In this regard, melon by-products and residues could be regarded as useful low-cost raw materials for the obtention and recovery of valued beneficial biomaterials with high market and industrial demand for the development of innovative starting ingredients or additives directed to the food (texturizers, softeners, and emulsifiers), cosmetic and medical industries (high water absorbing biomaterials and reinforcing and drug delivery systems). Therefore, the results obtained in this research showed that melon residues can be used as bioresources to obtain MCC, a natural biomaterial. As expected, the traditional process allowed for eliminating more precisely the non-cellulosic constituents, but using the thermo-alkaline process with a double-bleaching step allowed for better elimination of impurities, resulting in a purer MCC with a higher degree of crystallinity (61.94%) than the traditional process (54.80%). Overall, both methods allowed for the reduction of the lignocellulosic melon fibers to the microscale, as observed in the SEM micrographs. The microstructure with high porosity and the degree of crystallinity of the final white powder could highlight its potential applications as an additive in the formulation of novel biocomposites.

Therefore, the valorization and application of plant-based by-products as raw materials for the development of biomaterials can boost the transition to a zero-waste and circular economy, as well as reduce the high industrial dependence on synthetic plastics and their toxic components, avoiding environmental contamination and oil resource depletion. However, plant-based biomaterials are still under research, and further innovations and developments are needed. But, there is no doubt that cellulose-based materials are strongly emerging as key substitutes for traditional plastics, and they will become a reality in the next upcoming years.

## Figures and Tables

**Table 1:** Chemical composition of the melon-peel residues after pectin extraction.

Component (%) *	LMB	RHCl	RCA	RTA
Moisture	7.89 ± 0.51	11.12 ± 1.13	11.46 ± 0.46	12.16 ± 0.63
Total protein	8.80 ± 0.19	4.76 ± 0.24	2.77 ± 0.96	2.98 ± 0.13
Fat	1.74 ± 0.92	0.46 ± 0.16	0.67 ± 0.11	0.87 ± 0.47
Ash	1.67 ± 0.82	0.99 ± 0.11	1.16 ± 0.43	1.04 ± 0.19
Total carbohydrates **	79.90 ± 0.55	82.76 ± 1.29	83.94 ± 1.53	82.95 ± 1.89
Total fiber	37.10 ± 0.15	77.71 ± 0.56	49.60 ± 0.26	55.51 ± 0.34
Insoluble dietary fiber	35.51 ± 0.19	73.24 ± 0.45	46.67 ± 0.21	49.55 ± 0.47
Soluble dietary fiber	1.60 ± 0.10	4.47 ± 0.95	2.99 ± 1.33	5.96 ± 0.16

LMB: Lignocellulosic melon by-products; residues from HCl: hydrochloric acid; CA: citric acid; TA: tartaric acid. All determinations were carried out in triplicate and the mean value ± standard deviation. * Results are expressed in % of dry-matter basis (g/100 g DM). ** Total carbohydrate content was obtained by difference.

**Table 2:** Proximal structural composition of melon-peel residues after pectin extraction with different acid agents.

Component (g/100 g DM)	LMB	RHCl	RCA	RTA
Cellulose	24.24 ± 3.01	58.84 ± 6.00	40.13 ± 6.47	42.74 ± 7.03
Hemicellulose	11.04 ± 1.92	27.50 ± 2.24	21.33 ± 1.88	22.81 ± 3.08
Lignin	12.05 ± 1.53	29.46 ± 2.14	18.39 ± 0.72	20.4 ± 0.76

LMB: Lignocellulosic melon by-products; residues from HCl: hydrochloric acid; CA: citric acid; TA: tartaric acid; DM: dry matter. All determinations were carried out in triplicate and mean value ± standard.

**Table 3:** Mass yields (%) * obtained after each successive chemical treatment.

Step	Treatment					
	Thermo-Alkaline (2% NaOH-DB)			Traditional (4.5% NaOH)		
	RHCl	RCA	RTA	RHCl	RCA	RTA
Residues	39.21 ± 1.52	63.12 ± 3.88	57.83 ± 5.32	39.21 ± 1.53	63.12 ± 3.90	57.83 ± 5.34
Delignification	77.50 ± 4.21	83.54 ± 4.01	69.42 ± 5.41	53.66 ± 3.84	41.74 ± 5.31	40.18 ± 7.65
Bleaching	53.67 ± 1.94	49.81 ± 2.63	43.39 ± 1.25	71.59 ± 1.42	77.85 ± 3.73	77.04 ± 2.28
Acid hydrolysis (L-MCC)	13.04 ± 0.86	8.02 ± 0.53	10.15 ± 0.94	23.81 ± 1.07	19.23 ± 1.33	14.66 ± 1.75
Final yield	9.15 ± 2.87	4.76 ± 0.07	5.95 ± 0.01	2.58 ± 0.09	2.32 ± 0.10	3.29 ± 0.30

Residues after pectin extraction with HCl: hydrochloric acid; CA: citric acid; TA: tartaric acid; L-MCC: lyophilized microcrystalline cellulose; DB: double bleaching; Final yield is with respect to starting raw materials. * Results are expressed in % of dry-matter basis (g/100 g DM).

**Table 4:** Proximal structural composition of microcrystalline cellulose (MCC) from melon residues.

MCC Component (g/100 g DM)	Treatment					
	Thermo-Alkaline (2% NaOH-DB)			Traditional (4.5% NaOH)		
	RHCl	RCA	RTA	RHCl	RCA	RTA
Cellulose	48.34 ± 4.15	44.89 ± 6.19	46.31 ± 3.32	55.03 ± 3.12	51.40 ± 5.4	52.51 ± 4.61
Hemicellulose	27.21 ± 2.63	23.45 ± 3.87	25.83 ± 3.13	36.21 ± 1.36	29.20 ± 3.9	33.31 ± 5.18
Lignin	2.53 ± 1.18	3.54 ± 3.05	2.24 ± 5.45	1.76 ± 0.81	2.71 ± 0.32	1.84 ± 0.56
Ash	1.21 ± 0.66	2.22 ± 0.51	1.93 ± 0.25	0.94 ± 0.41	1.74 ± 0.77	1.32 ± 0.22
Moisture	4.43 ± 0.63	5.23 ± 0.72	4.10 ± 0.93	3.90 ± 0.21	4.14 ± 1.41	4.06 ± 1.08

Residues from HCl: hydrochloric acid; CA: citric acid; TA: tartaric acid; DB: double bleaching.

**Table 5:** Degree of crystallinity (%DC) of the melon samples treated with the different processes.

	Treatment (%DC)				
Sample		Hot-Acid Process (Residues)	Thermo-Alkaline (2% NaOH)	Thermo-Alkaline (2% NaOH-DB)	Traditional (4.5% NaOH)
LMB	31.82 ± 1.53				
MCC-HCl		47.75 ± 0.34	52.01 ± 0.30	61.94 ± 0.59	54.80 ± 5.58
MCC-CA		42.35 ± 0.53	49.72 ± 1.44	51.51 ± 0.97	54.87 ± 1.08
MCC-TA		44.13 ± 0.24	47.03 ± 1.92	56.53 ± 0.60	55.07 ± 0.41

LMB: lignocellulosic melon by-products; MCC: microcrystalline cellulose from HCl: hydrochloric acid; CA: citric acid; DB: double bleaching; MCC represents the final dried and milled samples.

## Data Availability

All data generated or analyzed during this study are included in this published article.
